# Reduction in extraocular muscle and orbital fat volumes after teprotumumab treatment in Japanese patients with thyroid eye disease: an exploratory magnetic resonance imaging analysis from the OPTIC-J trial

**DOI:** 10.1007/s10384-026-01372-x

**Published:** 2026-08-13

**Authors:** Ai Kozaki, Yasuhiro Takahashi, Yuji Tajiri, Zhenxun Wang, Tomoko Hayashida, Ada Kumar, Yuji Hiromatsu, Masashi Mimura

**Affiliations:** 1 Olympia Eye Hospital, 2-18-12 Jingumae, Shibuya-ku, Tokyo 1500001 Japan; 2 Department of Oculoplastic, Orbital & Lacrimal Surgery, Aichi Medical University Hospital, Nagakute, Aichi Japan; 3 Division of Endocrinology and Metabolism, Department of Medicine, Kurume University School of Medicine, Kurume, Fukuoka Japan; 4 Amgen Inc., Thousand Oaks, CA USA; 5 Diabetes, Thyroid and Endocrine Center, Shin Koga Hospital, Kurume, Fukuoka Japan; 6 Oculoplastic Surgery Center, Department of Ophthalmology, Osaka Kaisei Hospital, Yodogawa-ku, Osaka Japan

**Keywords:** Magnetic resonance imaging, Graves’ ophthalmopathy, Clinical trial, Imaging, Inflammation

## Abstract

**Purpose:**

This substudy of the OPTIC-J trial analyzed magnetic resonance imaging (MRI) scans from a subset of Japanese patients to examine changes in orbital tissues after teprotumumab treatment vs placebo for active thyroid eye disease (TED).

**Study design:**

Post hoc exploratory.

**Methods:**

Volumes and inflammation/edema of extraocular muscles (EOMs) and orbital fat were measured before and after 24 weeks of teprotumumab treatment or placebo using MRI in patients enrolled at four of the 16 trial sites. Inflammation/edema in EOM and orbital fat were evaluated using the signal intensity ratio, based on T2 intensity in the cerebral white matter, and an ordinal grading scale (0–3), respectively. Key clinical outcomes included proptosis, diplopia, and clinical activity score (CAS).

**Results:**

Mean (%) changes in total EOM and orbital fat volumes in study eyes treated with teprotumumab (*n* = 6) and placebo (*n* = 8) were –1,022 mm^3^ (–31%; *P* = 0.007) and –348 mm^3^ (–11%; *P* = 0.039), and –771 mm^3^ (–6%; *P* = 0.120) and 418 mm^3^ (4%; *P* = 0.084), respectively. Reductions were significantly greater with teprotumumab vs placebo (EOM, *P* = 0.021; fat, *P* = 0.016). Mean (%) changes in total EOM and orbital fat inflammation/edema grading of study eyes treated with teprotumumab were –1.82 (–31%; *P* = 0.005) and –1 (*P* = 0.031), respectively. Patients administered teprotumumab exhibited improvements in proptosis (83%), diplopia (67%), and resolution of activity determined by CAS (50%).

**Conclusion:**

In this OPTIC-J trial substudy, reductions in orbital soft tissue volumes and inflammation/edema were observed with MRI after teprotumumab treatment vs placebo; corresponding improvements in TED clinical outcomes were also observed.

**Supplementary Information:**

The online version contains supplementary material available at https://doi.org/10.1007/s10384-026-01372-x.

## Introduction

Thyroid eye disease (TED) is a clinically heterogenous, potentially disabling autoimmune disease associated with visual impairment and facial disfigurement [1, 2]. Although the pathogenesis of TED is not fully understood, it is linked to autoimmune responses targeting the insulin-like growth factor-1 receptor (IGF-1R) and/or the thyroid-stimulating hormone receptor (TSHR) present on orbital fibroblasts and thyrocytes [3]. Inflammation/edema and subsequent enlargement of the orbital soft tissues, including the extraocular muscles (EOMs) and orbital fat, can result in diplopia and proptosis [4, 5]. Severe soft tissue expansion may cause orbital apical crowding and subsequent dysthyroid optic neuropathy (DON), jeopardizing vision [1, 6, 7].

Teprotumumab, a fully human monoclonal antibody that blocks signaling through the IGF-1R complex with the TSHR, was the first TED treatment approved in the US and many other countries, including Japan [8–11]. Although teprotumumab has proven effective in improving inflammatory signs and symptoms—including proptosis, diplopia, and the clinical activity score (CAS) in patients with TED—specific effects of teprotumumab on orbital soft tissue volumes and inflammation/edema are unclear; additionally, previous trials did not compare teprotumumab versus placebo in patients with TED [2, 5, 12–14].

Due to variations in orbital anatomy, Asian patients with TED may present different clinical manifestations from non-Asian patients, including less prominent proptosis, potentially complicating assessment of disease activity/severity and delaying diagnosis. Efficacy and safety of teprotumumab in Japanese patients with TED were confirmed in a randomized, double-masked, placebo-controlled, phase 3 trial (OPTIC-J; jRCT2031210453; Japan registry of clinical trials) [5]. In this trial, 89% of patients treated with teprotumumab demonstrated improved proptosis by 2 mm or more after 24 weeks of treatment compared with 11% of patients receiving placebo (*P* < 0.0001) [11]. OPTIC-J was the first teprotumumab trial in Japanese patients with active TED and prospectively included magnetic resonance imaging (MRI) at four selected sites [5].

MRI and computed tomography (CT) are routinely used for the diagnosis and evaluation of TED in Japan [15, 16]. Although both CT and MRI can assess orbital soft tissue volume, MRI can more precisely evaluate EOM and orbital fat inflammation/edema [17]. Thus, MRI can be instrumental in determining TED activity in patients, and Japanese TED guidelines recommend basing the need for intervention on MRI findings [12, 18].

Here, we present exploratory MRI endpoints from a subset of OPTIC-J trial patients, including changes in EOM and orbital fat volumes and inflammation/edema within the orbital soft tissues with teprotumumab treatment versus placebo.

## Materials and methods

### Ethics

The original trial protocol was reviewed by the Pharmaceuticals and Medical Device Agency in Japan and approved by the Japanese Ministry of Health, Labour and Welfare and by the institutional review board or independent ethics committee of each institution participating in the primary trial of this post hoc analysis (CTN #2022-5099) [5]. The trial was conducted in accordance with the International Council for Harmonisation Good Clinical Practice guidelines and the principles of the Declaration of Helsinki. All patients provided written informed consent, including future publication of their medical images, prior to participating in this trial.

### Trial design

The parent OPTIC-J trial design was previously published [5]. Briefly, Japanese patients (20–80 years of age) with a diagnosis of Graves’ disease associated with TED, a study eye (the more proptotic eye) CAS ≥ 3 (out of 7), moderate-to-severe TED, an increase in proptosis ≥ 3 mm prior to TED diagnosis or proptosis ≥ 18 mm at randomization, and onset of TED within 9 months of baseline were enrolled. Those with prior orbital surgery for TED or DON were excluded from the trial. Eligible patients were randomized 1:1 to receive an intravenous infusion of teprotumumab or placebo once every 3 weeks for eight doses in this double-masked trial. Teprotumumab was initially administered at 10 mg/kg, and the remaining seven doses were administered at 20 mg/kg.

MRI was performed at four (of 16 total) preselected trial sites with patients from OPTIC-J at baseline (pretreatment) and week 24 (posttreatment). MRI of 1.5T or 3T scanner equipped with appropriate coils for orbital imaging was used to obtain images without enhancement, and the same equipment was used for all the images of each patient. Details for MRI equipment and protocols were standardized across the four sites. Images were transferred and measurements in both study and fellow eyes were performed by a masked central reader at Clario.

Neither patients nor the public were involved in the design, conduct, reporting, or dissemination of this substudy, besides those who participated as clinical trial patients/participants in the OPTIC-J parent study.

### Measurement of orbital soft tissue volumes by MRI

Volumes of total and individual EOMs and orbital fat were measured using three-dimensional volumetry on noncontrast T1-weighted MRI sequences on coronal sections. For each EOM, the cross-sectional area was measured in each slice where the EOM was visualized, and the total volume of EOMs was computed as the total summed area multiplied by slice separation (3 mm). Orbital fat volume was calculated similarly using the orbital cross-sectional area, excluding the optic nerve and EOM areas. Orbital tissue segmentation and volume calculations were performed by Clario using Image Workstation software version 10 (Clario) [5].

### Measurement of inflammation/edema by MRI

Inflammation/edema were measured in the EOMs and orbital fat using fat-suppressed T2-weighted coronal images. The signal intensity ratio (SIR) in each EOM was measured on two adjacent slices selected near the thickest part of the muscle belly and normalized to T2 intensity in the cerebral white matter (i.e., EOM signal divided by white matter signal and reported as the SIR). Orbital fat inflammation was graded based on the extent of elevated fluid signal within the fat of each orbit. A grading scale for inflammation in the orbital fat was defined as grade 0 (normal, no signs of inflammation), grade 1 (inflammation in ≤ 33% of the orbital fat), grade 2 (inflammation in 34%–66% of the orbital fat), or grade 3 (inflammation in ≥ 67% of the orbital fat).

### Measurement of clinical manifestations

Clinical manifestations were measured at each study visit. Proptosis was measured using a Hertel exophthalmometer. A proptosis response was defined as a reduction of ≥ 2 mm in the study eye without corresponding deterioration in the fellow eye. The same observer performed measurements at each visit to ensure consistency [19]. Diplopia was assessed using the Gorman score (0–3; 0 = no diplopia, 1 = intermittent diplopia [diplopia in primary position of gaze when tired or when first awakening], 2 = inconstant diplopia [diplopia at extremes of gaze], 3 = constant diplopia [continuous diplopia in primary or reading position]) [19]. A seven-point CAS assessment—including spontaneous/gaze-evoked orbital pain, eyelid swelling, conjunctival redness, eyelid erythema, chemosis, and inflammation of caruncle or plica—was performed. Eye motility, defined as the degree of restriction in the movement of the eye, was assessed at baseline and week 24 using the Hirschberg light reflex test with horizontal and vertical gaze instructions. Monocular ductions were recorded for supraduction, infraduction, adduction, and abduction.

### Statistical analysis

This post hoc analysis compared posttreatment changes in EOM and orbital fat volumes and orbital soft tissue inflammation/edema measured using MRI between the teprotumumab and placebo groups. Changes from baseline (CFBs) to week 24 in EOM volume, orbital fat volume, and SIR of EOM inflammation/edema were assessed by paired t-tests within each treatment group. The differences in CFBs at week 24 in EOM volume, orbital fat volume, and SIR of EOM inflammation/edema between teprotumumab and placebo were assessed by pooled t-tests. A Shapiro-Wilk test was conducted to test the normality assumption before applying t-tests, and no violations of normality assumptions were identified. The difference in CFB at week 24 in orbital fat inflammation/edema grading between the teprotumumab and placebo groups was assessed by a Wilcoxon two-sample exact test to determine nominal *P* values. All statistical tests were two-tailed, with *P* ≤ 0.05 set as the threshold for statistical significance. All *P* values were nominal and not adjusted for multiplicity.

## Results

### Baseline characteristics

Of 54 patients enrolled in the OPTIC-J trial, six patients who received teprotumumab and eight patients who received placebo, all with similar baseline characteristics, underwent MRI. The mean (SD) age of patients was 40.8 (11.1) and 47.6 (15.6) years in the teprotumumab and placebo groups, respectively. Mean (SD) proptosis at baseline was 18.8 (1.0) mm in the teprotumumab group and 20.0 (1.5) mm in the placebo group. Additional baseline characteristics are presented in Online Resource 1.

### EOM volume assessment

**Fig. 1:**
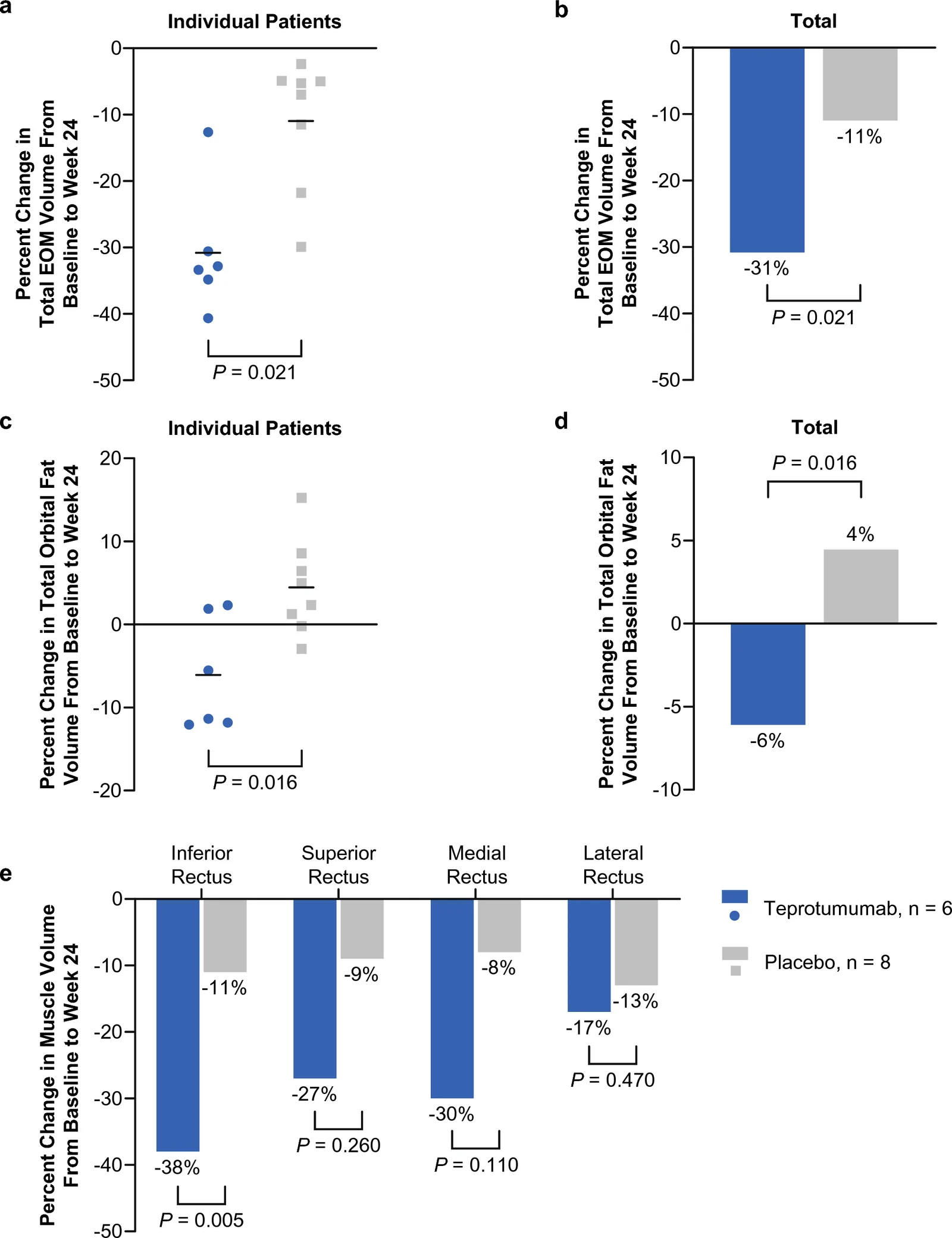
Posttreatment percent changes in total EOM volume by **a** individual patient and **b** total patient group, total orbital fat volume by **c** individual patient and **d** total patient group, and **e** each individual EOM volume in the study eyes. *EOM* extraocular muscle

The mean (SD) total EOM volumes at baseline in the teprotumumab and placebo groups were similar (Table 1 and Online Resource 2). At week 24, patients treated with teprotumumab showed significantly greater CFB reductions in total and individual EOM volumes in both the study and fellow eyes compared with placebo (Fig. 1, Table 1, and Online Resource 2). In the study eyes, the mean CFB at week 24 in total EOM volume was –1,022 mm^3^ (–31%; *P* = 0.007) and –348 mm^3^ (–11%; *P* = 0.039) with teprotumumab and placebo, respectively (Fig. 1 and Table 1). The reduction in EOM volume was significantly larger among patients receiving teprotumumab vs placebo (*P* = 0.021; Fig. 1).

**Table 1.** Change from baseline in volume of total and individual EOMs and orbital fat in the study eyes

	Teprotumumab (*n* = 6)					Placebo (*n* = 8)				
	Mean, mm^3^ (SD)			Percent change	*P* value^a^	Mean, mm^3^ (SD)			Percent change	*P* value^a^
	Baseline	Week 24	Volume difference			Baseline	Week 24	Volume difference		
Total EOM	3,085 (1,079)	2,062 (531)	–1,022 (563)	–31%	0.007	2,940 (717)	2,592 (568)	–348 (387)	–11%	0.039
Superior rectus muscle	686 (384)	458 (142)	–228 (262)	–27%	0.087	623 (199)	529 (118)	–94 (163)	–9%	0.150
Inferior rectus muscle	1,102 (512)	651 (258)	–451 (265)	–38%	0.009	913 (246)	814 (271)	–99 (105)	–11%	0.032
Medial rectus muscle	664 (228)	449 (114)	–215 (127)	–30%	0.009	702 (387)	621 (256)	–81 (156)	–8%	0.190
Lateral rectus muscle	633 (257)	504 (169)	–129 (147)	–17%	0.084	703 (141)	628 (237)	–75 (128)	–13%	0.140
Orbital fat volume	11,802 (4,350)	11,032 (3,766)	–771 (997)	–6%	0.120	12,939 (4,817)	13,357 (4,524)	418 (587)	4%	0.084

*EOM* extraocular muscle, *SD* standard deviation

^a^*P* values were calculated for volume change from day 1 to week 24 for four rectus muscles of the EOMs using a paired t-test

**Fig. 2:**
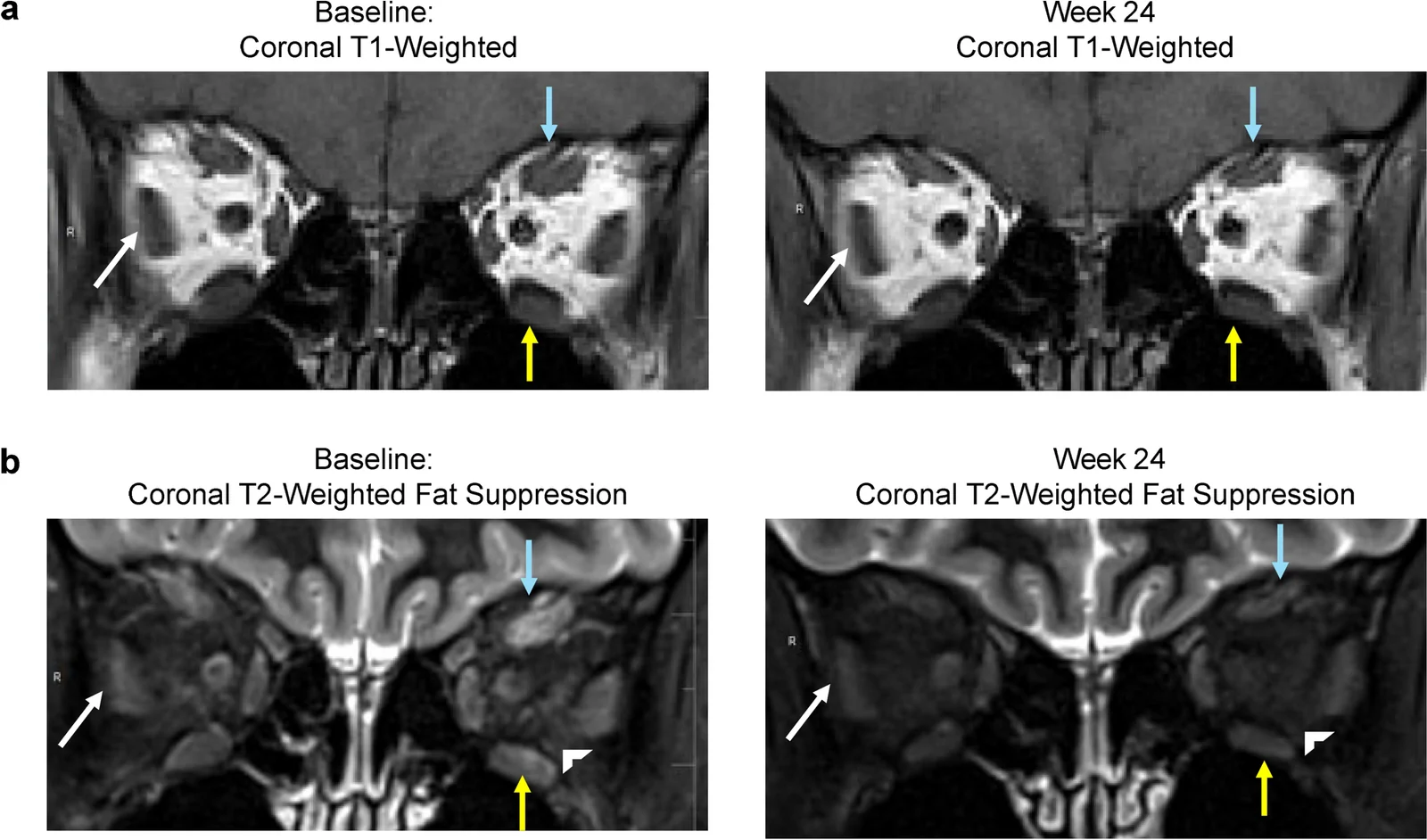
MRI scans taken at baseline and week 24 in a patient treated with teprotumumab demonstrating **a** reduction in the volume of orbital fat and EOMs with coronal T1-weighted MRI and **b** reduction in inflammation/edema of orbital fat and EOMs with T2-weighted fat-suppressed scans. Blue arrows indicate the superior rectus; yellow arrows indicate the inferior rectus; white arrows indicate the lateral rectus; and the white arrowhead indicates the orbital fat inflammation. *EOM* extraocular muscle, *MRI* magnetic resonance imaging

In the fellow eyes, the mean CFB at week 24 in total EOM volume was –526 mm^3^ (–21%; *P* = 0.017) in patients receiving teprotumumab and –246 mm^3^ (–9%; *P* = 0.031) in patients receiving placebo (Online Resource 2). While not statistically significant, the CFB reduction at week 24 in total EOM volume in the fellow eyes was numerically larger in patients receiving teprotumumab vs placebo (*P* = 0.12). Volumetric reductions of each EOM were also observed in both the study and fellow eyes and were numerically larger for teprotumumab vs placebo, as demonstrated in Table 1 and Online Resource 2, respectively. MRI taken at baseline and week 24 in a representative patient treated with teprotumumab are presented in Fig. 2.

### Orbital fat volume assessment

Mean (SD) total orbital fat volumes at baseline in the study eyes were 11,802 (4,350) mm^3^ with teprotumumab and 12,939 (4,817) mm^3^ with placebo. The mean CFB at week 24 in orbital fat volume in the study eyes was –771 mm^3^ (–6%; *P* = 0.120) and 418 mm^3^ (4%; *P* = 0.084) in the teprotumumab and placebo groups, respectively (Fig. 1 and Table 1). The reduction in orbital fat volume in the study eyes was significantly larger in patients receiving teprotumumab vs those receiving placebo (*P* = 0.016; Fig. 1). In the fellow eyes, the mean CFB at week 24 in orbital fat volume was –820 mm^3^ (–6%; *P* = 0.073) in patients receiving teprotumumab and 389 mm^3^ (4%; *P* = 0.300) in patients receiving placebo. The CFB at week 24 in orbital fat volume in the fellow eyes also demonstrated a significantly greater reduction with teprotumumab compared with placebo (*P* = 0.034).

### EOM inflammation/edema assessment

**Fig. 3:**
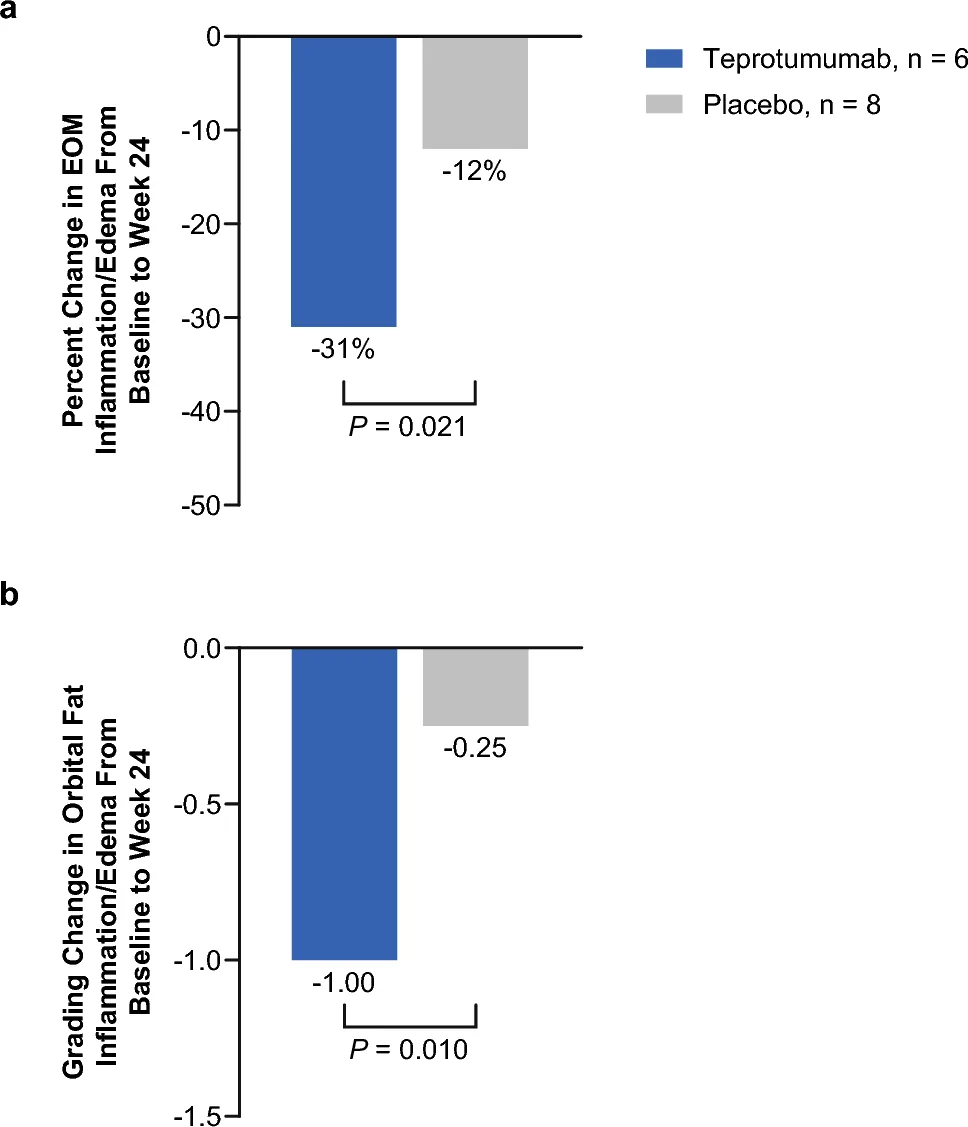
Comparison of posttreatment changes in **a** total EOM inflammation/edema and **b** total orbital fat inflammation/edema in the study eyes between the teprotumumab and placebo groups. *EOM* extraocular muscle

Patients receiving teprotumumab had a significant reduction in SIR in total EOM inflammation/edema at week 24 in the study eyes (mean CFB [%], –1.82 [–31%]; *P* = 0.005; Table 2). Patients receiving placebo also showed a significant, albeit less pronounced, CFB in SIR at week 24 in the study eyes (–0.73 [–12%]; *P* = 0.013; Table 2). When compared with those receiving placebo, patients receiving teprotumumab demonstrated a significant decrease in EOM inflammation/edema in the study eyes (*P* = 0.021; Fig. 3). A significant reduction at week 24 in total EOM inflammation/edema was observed in the fellow eyes of patients receiving teprotumumab (–0.88 [–17%]; *P* = 0.036), while a numerical reduction was seen in patients receiving placebo (–0.56 [–9%]; *P* = 0.060; Online Resource 2). A numerically larger decrease in EOM inflammation/edema in the fellow eyes was observed with teprotumumab vs placebo, although the difference did not reach statistical significance (*P* = 0.44).

**Table 2:** Comparisons in total EOM and total fat inflammation/edema between treatment groups in the study eyes

Baseline–week 24	Teprotumumab (*n* = 6)	*P* value	Placebo (*n* = 8)	*P* value
Total EOM inflammation/edema, mean (%)	–1.82 (–31%)	0.005	–0.73 (–12%)	0.013
Total fat inflammation/edema grading change, mean	–1.00	0.031	–0.25	0.50

*EOM* extraocular muscle

### Orbital fat inflammation/edema assessment

A significantly greater reduction at week 24 in orbital fat inflammation/edema grade was observed in the study eyes of patients who received teprotumumab (–1.00; *P* = 0.031) compared with the reduction observed with placebo (–0.25; *P* = 0.50; teprotumumab vs placebo, *P* = 0.010; Fig. 3 and Table 2). All six patients treated with teprotumumab achieved at least a one-grade improvement in orbital fat inflammation/edema in the study eyes and no changes in the fellow eyes. Comparatively, 25.0% (2/8) of patients who received placebo had at least a one-grade improvement in orbital fat inflammation/edema in the study eyes and no changes in the fellow eyes. Representative baseline and week 24 MRI scans depicting inflammation/edema in the EOMs and orbital fat are shown in Fig. 2.

### Clinical outcomes

**Fig. 4:**
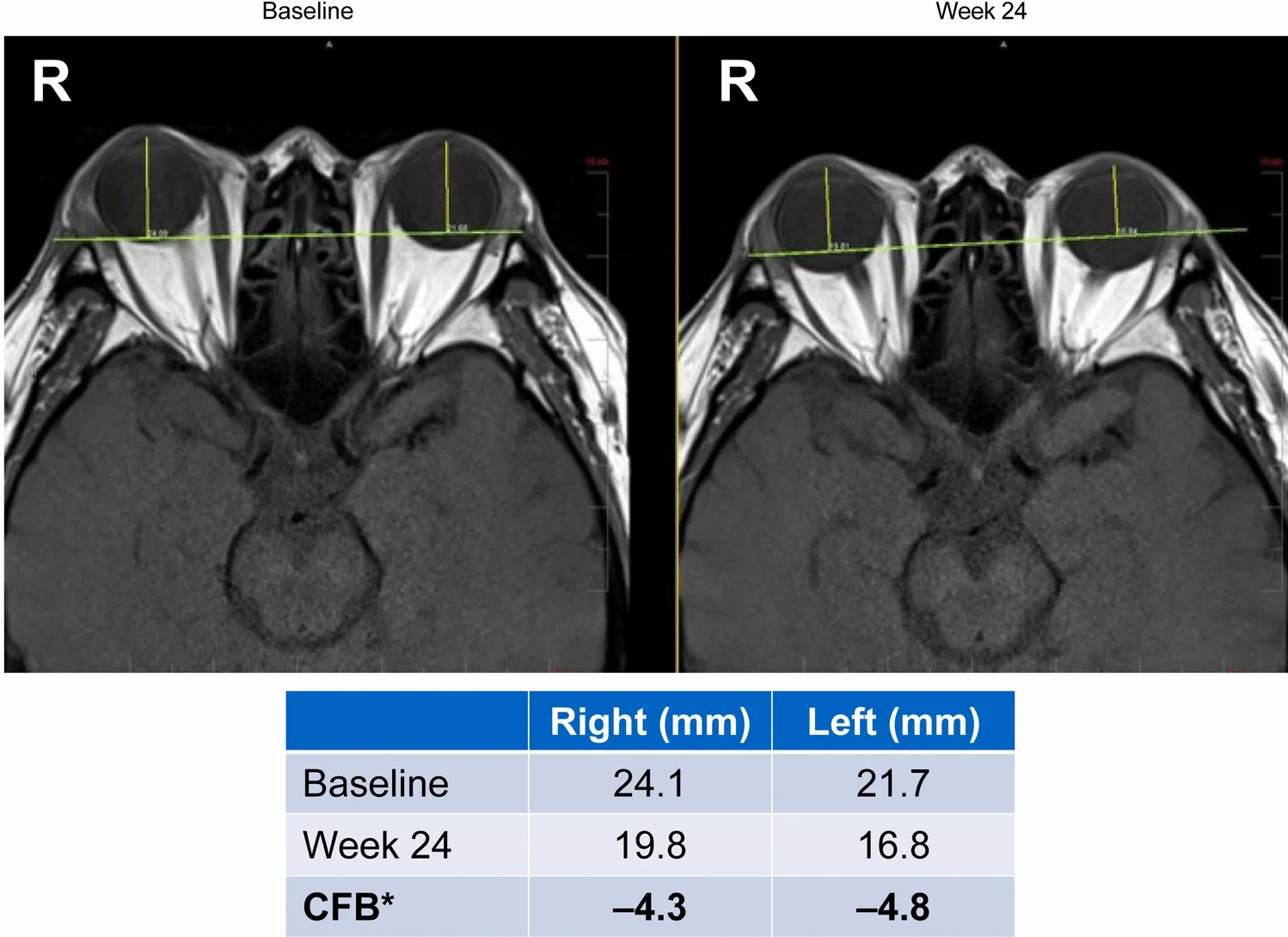
Confirmatory proptosis measurements of axial MRI scans for patient 4. *The CFB was calculated prior to rounding and may appear different than expected due to rounding. *CFB* change from baseline, *MRI* magnetic resonance imaging

Of patients who underwent MRI, 83.3% (5/6) of the teprotumumab group and 12.5% (1/8) of the placebo group were proptosis responders (≥ 2 mm reduction in proptosis in the study eye). Study eye proptosis measurements are in Online Resource 3. Of note, screening and baseline proptosis measurements for patient 4 of the teprotumumab group were incorrectly performed, which was discovered at week 3 when the correctly performed measurement was noticeably higher (≥ 3 mm in the study eye), leading to ostensive worsening of the proptosis. Therefore, ad hoc proptosis measurement by MRI was conducted for only this patient to confirm proptosis response from baseline to week 24 (study orbit CFB, –4.3; fellow orbit CFB, –4.8; Fig. 4). Pretreatment and posttreatment MRI showed an apparent reduction in proptosis, EOM volume, and orbital fat volume. At baseline, 100% (6/6) of the teprotumumab-treated patients had diplopia (one intermittent, four inconstant, and one constant), and 88% (7/8) of placebo-treated patients had diplopia (four inconstant and three constant). Diplopia response was achieved in 66.7% (4/6) of teprotumumab-treated patients. In the placebo group, 42.9% (3/7) of patients had a diplopia response while 14.3% (1/7) had worsening. Disease inactivation (CAS of 0 or 1) was achieved in 50.0% (3/6) of teprotumumab-treated patients, with a mean change of –2.3, and 12.5% (1/8) of placebo-treated patients, with a mean change of –1.5. All six patients treated with teprotumumab had an improved CAS from baseline. Clinical measurement outcomes in the study and fellow eyes of individual patients are listed in Online Resources 3 and 4, respectively. A degree of heterogeneity was observed in EOM and orbital fat volumes (Online Resource 3). One patient had impaired motility in all four ductions and showed improvement at week 24, as seen in Online Resource 5 (fellow eye, Online Resource 6).

## Discussion

The subset of patients with TED who participated in the OPTIC-J trial and underwent pre- and posttreatment MRI scans at one of four preselected trial sites demonstrated significant reductions in EOM and orbital fat volumes in the study eyes with teprotumumab compared with placebo. Furthermore, numerically and significantly greater reductions were observed in EOM and orbital fat volumes, respectively, in the fellow eyes in the teprotumumab group compared with the placebo group. Patients receiving teprotumumab also demonstrated statistically significant reductions in inflammation/edema of EOMs and orbital fat in the study eyes compared with those receiving placebo.

IGF-1R, a receptor overexpressed in orbital fibroblasts in TED, forms a complex with TSHR. Binding of autoantibodies to this complex leads to inflammation, cytokine and hyaluronan production, and adipogenesis, resulting in soft tissue expansion and inflammation/edema—the hallmark mechanisms of proptosis and diplopia in TED [5, 9, 20–22]. Though not well understood, improvements in proptosis and diplopia are thought to result from teprotumumab blocking downstream signaling via the IGF-1R–TSHR complex [9, 22].

Reduced EOM and orbital fat volumes were observed in the MRI scans of patients treated with teprotumumab [23]. However, none of those previously reported observations included data from patients treated with placebo. MRI showed a significant decrease in T2 SIR in the EOMs and orbital fat among patients receiving teprotumumab vs those receiving placebo. Similar to other studies, the orbital soft tissue remodeling observed in patients with TED treated with teprotumumab was likely associated with the improvement in proptosis and the fact that there was no worsening of diplopia following teprotumumab treatment [5, 24]. As baseline diplopia scores varied between patients receiving teprotumumab and placebo in the current study, future studies are needed to ensure similar baseline diplopia between treatment groups to make direct comparisons. Although motility impairment was not extensive at baseline for patients in this substudy, baseline supraduction was impaired in all six patients treated with teprotumumab; at week 24, noticeable improvement among the three patients with the most severe baseline supraduction impairment was observed, which corresponded specifically to the large improvement in the inferior rectus muscle volume, swelling or hardening of which contributes to restricted supraduction. One teprotumumab-treated patient had increased proptosis at week 24 due to an incorrect baseline measurement, which presumably was lower-than-actual proptosis. Notably, MRI confirmed improvement in proptosis, reductions in total EOM and orbital fat volumes, and improvement in orbital inflammation/edema in this patient. These data indicate improvement in orbital soft tissue inflammation/edema in patients treated with teprotumumab in contrast to placebo. This improvement may also reflect the improvement in CAS at week 24 from baseline observed in all six patients treated with teprotumumab.

The OPTIC-J trial uniquely presents the efficacy of teprotumumab among Asian patients with active TED, as this population is distinct from previous trials that included mostly White populations [2]. Furthermore, this substudy of the OPTIC-J trial presents the first MRI evidence of teprotumumab’s activity on the retro-orbital EOMs and fat in a Japanese population. Due to differences in anatomical orbital structures, proptosis may be less severe in Asian vs European or Western populations. The upper limit of the normal range for Hertel exophthalmometry measurements in White patients is 21 mm but only 17.7 mm or 18.3 mm in Japanese or Korean populations [25, 26]. While less severe proptosis complicates accurate assessment of disease activity/severity, doctors should carefully evaluate milder proptosis in Asian patients as these patients sometimes present with inflammation/edema in EOMs and orbital fat, leading to DON without apparent proptosis [6, 25]. The MRI findings in this OPTIC-J post hoc analysis demonstrate for the first time an understanding of the effect of teprotumumab on orbital soft tissue remodeling in Asian patients with TED. Similar to the findings observed in the OPTIC trial [2], in which MRI was performed in a small subset of predominantly White patients at a single participating site without a placebo group, our results show significant reductions in EOM and orbital fat volumes and orbital soft tissue inflammation/edema—as well as improvements in proptosis, diplopia, and CAS—following teprotumumab treatment.

Limitations of this post hoc analysis include the small sample size. Out of 16 sites that participated in the parent OPTIC-J trial, four sites were preselected to perform MRI, and patients enrolled to the parent trial at these preselected sites underwent MRI. Of note, to ensure unbiased selection, these patients were randomized to teprotumumab or placebo in a double-masked manner in tandem with patients at all 16 sites. The results were suggestive of the overall OPTIC-J population with a comparable improvement in clinical outcomes between OPTIC-J vs the subgroup herein who underwent MRI (proptosis responder rate, 89% vs 83%; diplopia resolution, 50% vs 67%; CAS complete responder rate, 59% vs 50%) [5, 14, 17]. Due to the limited number of patients in the current substudy, analyses correlating MRI and clinical outcomes were inconclusive. Therefore, we believe imaging analysis of patients in the OPTIC-J trial elucidates orbital soft tissue impact in TED and teprotumumab treatment effects [5]. Together, these data offer a more comprehensive picture of teprotumumab’s generalizability and efficacy in diverse patient populations. Another limitation of this study was the short trial follow-up period (30 days) and study period of only 24 weeks. However, analyses in previous trials show that reduction of proptosis was maintained in the majority of patients 1 year post-teprotumumab therapy, indicating that teprotumumab has the potential to provide long-term clinical improvement [5, 27, 28]. The OPTIC-J trial included only patients with active TED of short duration and high activity; however, in an additional trial conducted in patients with chronic TED (duration 2–10 years) and low activity, MRI assessment of patients also showed reductions in EOM and orbital fat volume after teprotumumab [10]. Finally, as the OPTIC-J trial excluded patients with prior orbital surgery and DON, future studies are needed to better understand the impact of teprotumumab on volumetric and inflammatory changes in the EOMs and orbital fat in patients with TED with a history of orbital decompression and/or DON.

Teprotumumab treatment resulted in significant reductions in EOM and orbital fat volumes in study eyes of patients with TED as well as improvements in orbital soft tissue inflammation/edema compared with placebo. Accordingly, patients receiving teprotumumab showed improved clinical outcomes, including proptosis, diplopia, and CAS. Using MRI in Japanese patients with TED allows for an improved understanding of the treatment impact of teprotumumab with disease monitoring of the orbital soft tissues in this population.

## Supplementary information

Below is the link to the electronic supplementary material.

Supplementary file1 (DOCX 109 KB)

## Data Availability

Qualified researchers may request data from Amgen clinical studies. Complete details are available at the following link: https://www.amgen.com/science/clinical-trials/clinical-data-transparency-practices/clinical-trial-data-sharing-request.
