# Supplementary material for: Reduction in extraocular muscle and orbital fat volumes after teprotumumab treatment in Japanese patients with thyroid eye disease: an exploratory magnetic resonance imaging analysis from the OPTIC-J trial
Source: Jpn J Ophthalmol. 2026 Aug 13;70(5):1089–98. doi: 10.1007/s10384-026-01372-x (PMC13615915; doi:10.1007/s10384-026-01372-x)
Supplement: Supplementary file 1 [file 10384_2026_1372_MOESM1_ESM.docx]

**Supplemental material**

**Online Resource 1.** Patient characteristics at baseline

|  | **Teprotumumab**  (n = 6) | **Placebo**  (n = 8) |
| --- | --- | --- |
| **Sex, n (%)**  Female | 5 (83.3) | 6 (75.0) |
| **Age, years, mean** | 40.8 | 47.6 |
| **Proptosis in study eye, mm, mean** | 18.8 | 20.0 |
| **Diplopia (Gorman score), n (%)**  0 (no diplopia)  1 (intermittent)  2 (inconstant)  3 (constant) | 0  1 (16.7)  4 (66.7)  1 (16.7) | 1 (12.5)  0  4 (50.0)  3 (37.5) |
| **CAS in study eye, mean (SD)** | 3.5 (0.5) | 3.8 (0.7) |

CAS, clinical activity score; SD, standard deviation

**Online Resource 2. (a)** Posttreatment changes in total EOM, individual EOM, and orbital fat volumes and **(b)** total EOM and total fat inflammation/edema in the fellow eyes

**a**

|  | **Teprotumumab (n = 6)** | | | | **Placebo (n = 8)** | | | |
| --- | --- | --- | --- | --- | --- | --- | --- | --- |
|  | **Baseline**  **(mm^3^, mean [SD])** | **Week 24**  **(mm^3^, mean [SD])** | **Volume change, mm^3^, mean (% change)** | ***P* value^a^** | **Baseline**  **(mm^3^, mean [SD])** | **Week 24**  **(mm^3^, mean [SD])** | **Volume change, mm^3^, mean (% change)** | ***P* value^a^** |
| **Total EOM volume** | 2,332  (729) | 1,806  (414) | –526  (–21%) | 0.017 | 2,552  (677) | 2,306  (554) | –246  (–9%) | 0.031 |
| **Superior rectus muscle** | 513  (230) | 373  (105) | –140  (-24%) | 0.075 | 571  (198) | 500  (165) | –71  (-10%) | 0.140 |
| **Inferior rectus muscle** | 699  (292) | 548  (148) | –150  (-18%) | 0.096 | 790  (253) | 720  (200) | –69  (-7%) | 0.100 |
| **Medial rectus muscle** | 568  (226) | 451  (116) | –116  (-17%) | 0.070 | 578  (131) | 531  (108) | –47  (-7%) | 0.053 |
| **Lateral rectus muscle** | 552  (162) | 433  (119) | –119  (-20%) | 0.018 | 613  (223) | 555  (165) | –58  (-7%) | 0.054 |
| **Orbital fat volume** | 11,287  (3,593) | 10,467  (2,831) | –820  (–6%) | 0.073 | 12,659  (4,129) | 13,047  (3,928) | 389  (4%) | 0.30 |

**b**

| **Baseline–week 24** | **Teprotumumab**  **(n = 6)** | ***P* value** | **Placebo**  **(n = 8)** | ***P* value** |
| --- | --- | --- | --- | --- |
| **Total EOM inflammation/edema, mean (%)** | –0.88 (–17%) | 0.036 | –0.56 (–9%) | 0.060 |
| **Total fat inflammation/edema grading change, mean** | 0 | N/A | 0 | N/A |

^a^*P* values were calculated for volume change from day 1 to week 24 for four rectus muscles of the EOMs using a paired *t* test

EOM, extraocular muscle; N/A, not available; SD, standard deviation

**Online Resource 3.** Treatment outcomes in individual patients after receiving teprotumumab or placebo in the study eye

|  | **Teprotumumab (n = 6)** | | | | |
| --- | --- | --- | --- | --- | --- |
|  | **Proptosis,**  **mm** | **Diplopia,**  **Gorman score** | **CAS** | **Total EOM volume, mm^3^, mean**  **(% change)** | **Total orbital fat volume, mm^3^, mean (% change)** |
| **Patient 1**  Baseline  Week 24  CFB (%) | 18.0  15.5  –2.5 | 3  2  –1 | 3  0  –3 | 4,616  2,741  –1,875 (–41%) | 8,336  7,330  –1,005 (–12%) |
| **Patient 2**  Baseline  Week 24  CFB (%) | 17.5  15.0  –2.5 | 2  2  0 | 4  2  –2 | 2,864  1924  –940 (–33%) | 9,434  8,911  –523 (–6%) |
| **Patient 3**  Baseline  Week 24  CFB (%) | 19.0  16.0  –3.0 | 2  1  –1 | 4  3  –1 | 1,625  1,420  –205 (–13%) | 10,200  9,042  –1,158 (–11%) |
| **Patient 4**  Baseline  Week 24  CFB (%) | 18.5  23.0  4.5 | 1  0  –1 | 3  2  –1 | 3,960  2,639  –1,321 (–33%) | 20,367  17,959  –2,408 (–12%) |
| **Patient 5**  Baseline  Week 24  CFB (%) | 20.0  18.0  –2.0 | 2  2  0 | 4  0  –4 | 2,353  1,634  –719 (–31%) | 11,692  11,912  221 (2%) |
| **Patient 6**  Baseline  Week 24  CFB (%) | 20.0  17.0  –3.0 | 2  0  –2 | 3  0  –3 | 3,089  2014  –1,075 (–35%) | 10,785  11,035  250 (2%) |
|  | **Placebo (n = 8)** | | | | |
|  | **Proptosis,**  **mm** | **Diplopia,**  **Gorman score** | **CAS** | **Total EOM volume, mm^3^, mean**  **(% change)** | **Total orbital fat volume, mm^3^, mean (% change)** |
| **Patient 1**  Baseline  Week 24  CFB (%) | 18.5  19.0  0.5 | 2  3  1 | 5  3  –2 | 3,543  3,366  –177 (–5%) | 20,674  20,633  –41 (0%) |
| **Patient 2**  Baseline  Week 24  CFB (%) | 21.0  19.0  –2.0 | 2  2  0 | 3  3  0 | 2,141  2029  –113 (–5%) | 10,808  10,942  134 (1%) |
| **Patient 3**  Baseline  Week 24  CFB (%) | 19.0  19.0  0 | 2  1  –1 | 3  1  –2 | 2,843  2,775  –68 (–2%) | 8,602  8,802  200 (2%) |
| **Patient 4**  Baseline  Week 24  CFB (%) | 18.5  20.0  1.5 | 3  3  0 | 4  3  –1 | 3,532  3,285  –247 (–7%) | 14,243  14,951  708 (5%) |
| **Patient 5**  Baseline  Week 24  CFB (%) | 20.0  19.0  –1.0 | 3  2  –1 | 3  2  –1 | 2,451  1918  –533 (–22%) | 9,763  10,391  628 (6%) |
| **Patient 6**  Baseline  Week 24  CFB (%) | 22.0  22.0  0 | 3  3  0 | 4  2  –2 | 4,124  2,892  –1,233 (–30%) | 13,881  15,072  1,192 (9%) |
| **Patient 7**  Baseline  Week 24  CFB (%) | 22.0  21.0  –1.0 | 2  0  –2 | 4  2  –2 | 2,673  2,366  –307 (–11%) | 7,003  8,070  1,067 (15%) |
| **Patient 8**  Baseline  Week 24  CFB (%) | 19.0  18.0  –1.0 | 0  0  0 | 4  2  –2 | 2,215  2,107  –108 (–5%) | 18,538  17,992  –546 (–3%) |

CAS, clinical activity score; CFB, change from baseline; EOM, extraocular muscle

**Online Resource 4.** Treatment outcomes in the fellow eye in individual patient after receiving teprotumumab or placebo

|  | **Teprotumumab (n = 6)** | | | | |
| --- | --- | --- | --- | --- | --- |
|  | **Proptosis,**  **mm** | **Diplopia,**  **Gorman score** | **CAS** | **Total EOM volume,**  **mm^3^, mean**  **(% change)** | **Total orbital fat volume, mm^3^, mean (% change)** |
| **Patient 1**  Baseline  Week 24  CFB (%) | 16.5  14.5  –2.0 | 3  2  -1 | 3  0  –3 | 3,428  2,287  –1,140 (–33%) | 8,421  8,290  –131 (–2%) |
| **Patient 2**  Baseline  Week 24  CFB (%) | 13.5  13.0  –0.5 | 2  2  0 | 4  3  –1 | 2,604  1,855  –749 (–29%) | 10,259  9,741  –518 (–5%) |
| **Patient 3**  Baseline  Week 24  CFB (%) | 18.0  16.0  –2.0 | 2  1  -1 | 4  3  –1 | 1,773  1,513  –260 (–15%) | 10,702  9,517  –1,185 (–11%) |
| **Patient 4**  Baseline  Week 24  CFB (%) | 18.0  22.0  4.0 | 1  0  -1 | 3  2  –1 | 2,776  2,242  –534 (–19%) | 18,286  16,118  –2,168 (–12%) |
| **Patient 5**  Baseline  Week 24  CFB (%) | 18.0  16.0  –2.0 | 2  2  0 | 1  0  –1 | 1,498  1,224  –275 (–18%) | 8,847  9,148  301 (3%) |
| **Patient 6**  Baseline  Week 24  CFB (%) | 17.0  16.0  –1.0 | 2  0  -2 | 0  0  0 | 1912  1,716  –196 (–10%) | 11,209  9,989  –1,220 (–11%) |
|  | **Placebo (n = 8)** | | | | |
|  | **Proptosis,**  **mm** | **Diplopia,**  **Gorman score** | **CAS** | **Total EOM volume,**  **mm^3^, mean**  **(% change)** | **Total orbital fat volume, mm^3^, mean (% change)** |
| **Patient 1**  Baseline  Week 24  CFB (%) | 13.5  14.0  0.5 | 2  3  1 | 1  1  0 | 2,168  2050  –118 (–5%) | 17,947  16,991  –956 (–5%) |
| **Patient 2**  Baseline  Week 24  CFB (%) | 16.5  15.0  –1.5 | 2  2  0 | 5  2  –3 | 1,847  1941  94 (5%) | 11,288  11,207  –81 (–1%) |
| **Patient 3**  Baseline  Week 24  CFB (%) | 18.0  19.0  1.0 | 2  1  -1 | 3  1  –2 | 2,394  2,107  –287 (–12%) | 8,374  8,562  188 (2%) |
| **Patient 4**  Baseline  Week 24  CFB (%) | 18.0  19.0  1.0 | 3  3  0 | 4  2  –2 | 3,677  3,413  –265 (–7%) | 14,147  15,492  1,346 (10%) |
| **Patient 5**  Baseline  Week 24  CFB (%) | 18.0  18.0  0 | 3  2  -1 | 1  1  0 | 1,633  1,577  –56 (–3%) | 9,233  8,847  –385 (–4%) |
| **Patient 6**  Baseline  Week 24  CFB (%) | 21.0  22.0  1.0 | 3  3  0 | 3  1  –2 | 2,875  2,691  –185 (–6%) | 13,726  15,255  1,529 (11%) |
| **Patient 7**  Baseline  Week 24  CFB (%) | 20.0  20.0  0 | 2  0  -2 | 4  2  –2 | 3,090  2,318  –773 (–25%) | 8,019  9,620  1,601 (20%) |
| **Patient 8**  Baseline  Week 24  CFB (%) | 19.0  18.0  –1.0 | 0  0  0 | 4  2  –2 | 2,733  2,353  –380 (–14%) | 18,534  18,403  –131 (–1%) |

CAS, clinical activity score; CFB, change from baseline; EOM, extraocular muscle

**Online Resource 5.** Posttreatment changes in extraocular motility in individual patients in the study eyes

|  | **Baseline (degrees)** | | | | **Week 24 (degrees)** | | | |
| --- | --- | --- | --- | --- | --- | --- | --- | --- |
|  | **Supraduction^a^** | **Infraduction^a^** | **Adduction^a^** | **Abduction^a^** | **Supraduction^a^** | **Infraduction^a^** | **Adduction^a^** | **Abduction^a^** |
| **Teprotumumab (n = 6)** | | | | | | | | |
| Patient 1 | 20 | 45 | 45 | 40 | 15 | 45 | 45 | 45 |
| Patient 2 | 30 | 45 | 45 | 45 | 30 | 45 | 45 | 45 |
| Patient 3 | 15 | 45 | 45 | 45 | 20 | 45 | 30 | 45 |
| Patient 4 | 15 | 30 | 30 | 15 | 30 | 45 | 45 | 45 |
| Patient 5 | 45 | 45 | 45 | 45 | 40 | 45 | 45 | 45 |
| Patient 6 | 15 | 45 | 45 | 45 | 40 | 45 | 45 | 45 |
| **Placebo (n = 8)** | | | | | | | | |
| Patient 1 | 15 | 45 | 45 | 45 | 15 | 40 | 35 | 45 |
| Patient 2 | 15 | 45 | 45 | 45 | 15 | 45 | 45 | 45 |
| Patient 3 | 45 | 45 | 45 | 45 | 45 | 45 | 45 | 45 |
| Patient 4 | 40 | 45 | 45 | 30 | 30 | 45 | 45 | 30 |
| Patient 5 | 15 | 45 | 45 | 45 | 15 | 45 | 45 | 45 |
| Patient 6 | 15 | 45 | 45 | 30 | 20 | 45 | 45 | 30 |
| Patient 7 | 30 | 30 | 45 | 45 | 30 | 30 | 45 | 45 |
| Patient 8 | 30 | 45 | 30 | 45 | 30 | 45 | 30 | 45 |

^a^A score of 45 indicates no issues

**Online Resource 6.** Posttreatment changes in extraocular motility in individual patients in the fellow eyes

|  | **Baseline (degrees)** | | | | **Week 24 (degrees)** | | | |
| --- | --- | --- | --- | --- | --- | --- | --- | --- |
|  | **Supraduction^a^** | **Infraduction^a^** | **Adduction^a^** | **Abduction^a^** | **Supraduction^a^** | **Infraduction^a^** | **Adduction^a^** | **Abduction^a^** |
| **Teprotumumab (n = 6)** | | | | | | | | |
| Patient 1 | 15 | 45 | 45 | 45 | 20 | 45 | 45 | 45 |
| Patient 2 | 35 | 45 | 45 | 45 | 30 | 45 | 45 | 45 |
| Patient 3 | 20 | 45 | 45 | 45 | 20 | 45 | 45 | 45 |
| Patient 4 | 30 | 30 | 30 | 30 | 30 | 45 | 45 | 45 |
| Patient 5 | 15 | 45 | 45 | 45 | 45 | 45 | 45 | 45 |
| Patient 6 | 30 | 45 | 45 | 45 | 45 | 45 | 45 | 45 |
| **Placebo (n = 8)** | | | | | | | | |
| Patient 1 | 45 | 45 | 45 | 45 | 45 | 45 | 45 | 45 |
| Patient 2 | 35 | 45 | 45 | 45 | 25 | 45 | 45 | 45 |
| Patient 3 | 30 | 45 | 45 | 45 | 45 | 45 | 45 | 45 |
| Patient 4 | 40 | 45 | 45 | 30 | 45 | 45 | 45 | 45 |
| Patient 5 | 30 | 45 | 45 | 45 | 30 | 45 | 45 | 45 |
| Patient 6 | 30 | 45 | 45 | 45 | 20 | 45 | 45 | 45 |
| Patient 7 | 30 | 30 | 45 | 45 | 30 | 30 | 45 | 45 |
| Patient 8 | 30 | 45 | 30 | 45 | 30 | 45 | 30 | 45 |

^a^A score of 45 indicates no issues
